# Fast and Slow Inhibition in the Visual Thalamus Is Influenced by Allocating GABA_A_ Receptors with Different γ Subunits

**DOI:** 10.3389/fncel.2017.00095

**Published:** 2017-04-04

**Authors:** Zhiwen Ye, Xiao Yu, Catriona M. Houston, Zahra Aboukhalil, Nicholas P. Franks, William Wisden, Stephen G. Brickley

**Affiliations:** ^1^Department of Life Sciences, Imperial College LondonLondon, UK; ^2^Department of Neurophysiology, The Francis Crick InstituteLondon, UK

**Keywords:** GABA, synapse, thalamus, inhibition

## Abstract

Cell-type specific differences in the kinetics of inhibitory postsynaptic conductance changes (IPSCs) are believed to impact upon network dynamics throughout the brain. Much attention has focused on how GABA_A_ receptor (GABA_A_R) α and β subunit diversity will influence IPSC kinetics, but less is known about the influence of the γ subunit. We have examined whether GABA_A_R γ subunit heterogeneity influences IPSC properties in the thalamus. The γ2 subunit gene was deleted from GABA_A_Rs selectively in the dorsal lateral geniculate nucleus (dLGN). The removal of the γ2 subunit from the dLGN reduced the overall spontaneous IPSC (sIPSC) frequency across all relay cells and produced an absence of IPSCs in a subset of relay neurons. The remaining slower IPSCs were both insensitive to diazepam and zinc indicating the absence of the γ2 subunit. Because these slower IPSCs were potentiated by methyl-6,7-dimethoxy-4-ethyl-β-carboline-3-carboxylate (DMCM), we propose these IPSCs involve γ1 subunit-containing GABA_A_R activation. Therefore, γ subunit heterogeneity appears to influence the kinetics of GABA_A_R-mediated synaptic transmission in the visual thalamus in a cell-selective manner. We suggest that activation of γ1 subunit-containing GABA_A_Rs give rise to slower IPSCs in general, while faster IPSCs tend to be mediated by γ2 subunit-containing GABA_A_Rs.

## Introduction

The dorsal lateral geniculate nucleus (dLGN) transmits visual information from the retina to the visual cortex (Nassi and Callaway, 2009) with a variety of modulatory inputs influencing how this information is processed; including glutamatergic cortical inputs, cholinergic brain stem inputs and GABAergic inputs (Sherman and Guillery, 2002; Saalmann and Kastner, 2011). GABAergic modulation originates from both local interneurons within the dLGN (Rafols and Valverde, 1973; Ohara et al., 1983; Acuna-Goycolea et al., 2008; Seabrook et al., 2013) and external projections from the thalamic reticular nucleus (nRT; Sumitomo et al., 1976; Montero and Scott, 1981). These inputs can shape receptive field properties (Sillito and Kemp, 1983; Norton and Godwin, 1992) and regulate visual attention (Hirsch et al., 2015; Wimmer et al., 2015) through the activation of GABA_A_ and GABA_B_ receptors.

GABA_A_ receptor (GABA_A_R) heterogeneity is particularly influential in generating the variability in inhibitory postsynaptic conductance (IPSC) kinetics that shapes network behavior in the brain. Synaptic GABA_A_Rs are assembled from α, β and γ subunits (Olsen and Sieghart, 2009). Each α subunit (α1 to α6) produces a particular kinetics with a decay of only a few milliseconds for α1 subunit-containing GABA_A_Rs (Bartos et al., 2001), tens of milliseconds for α3 subunit-containing GABA_A_Rs (Eyre et al., 2012) and around a 100 ms for the slow component of the IPSC mediated by α6 subunit-containing GABA_A_Rs (Bright et al., 2011). The β subunit (β1 to β3) has a more subtle influence on IPSC kinetics related to the phosphorylation status of the β subunit (Nusser et al., 1998; Houston et al., 2009).

Three γ subunits (γ1 to γ3) exist (Pritchett et al., 1989; Ymer et al., 1990; Herb et al., 1992), but the importance of γ subunit variability for IPSC kinetics has been little considered because γ2 subunit expression dominates in most brain regions (Wisden et al., 1992; Pirker et al., 2000). The global γ2 gene knockout is lethal (Günther et al., 1995), and the γ2 subunit appears essential for targeting of GABA_A_Rs to the synapse and the generation of fast IPSCs (Essrich et al., 1998; Schweizer et al., 2003; Wulff et al., 2007, 2009b), but the absence of IPSCs in the γ2 knockout mice can be rescued with γ3 gene overexpression (Baer et al., 1999). By contrast, the γ1 subunit produces a looser clustering of GABA_A_Rs at synapses and, therefore, results in the generation of slower IPSCs (Dixon et al., 2014).

Genetically deleting the γ2 subunit removes all IPSCs from Purkinje cells (Wulff et al., 2009b), ventrobasal (VB) thalamic relay neurons (Rovó et al., 2014), hippocampal parvalbumin interneurons (Wulff et al., 2009a) and histaminergic hypothalamic neurons (Zecharia et al., 2012), as well as massively reducing IPSC amplitude and frequency in hypothalamic GnRH neurons (Lee et al., 2010). Similarly, in some neocortical neurons γ2 gene ablation reduces IPSC frequency and in this case the γ3 subunit appears to cluster the remaining GABA_A_Rs (Kerti-Szigeti et al., 2014). Here, we report that removal of the γ2 subunit from the dLGN removes IPSCs from only half of the relay neurons and we provide pharmacological evidence that the remaining slower IPSCs are most likely mediated by γ1 subunit-containing GABA_A_Rs.

## Materials and Methods

### Mouse Strains

The HDC-Cre line was generated by homologous recombination with an ires-Cre cassette inserted into exon 12 of the hdc gene, between the stop (TAG) codon and the polyadenylation (pA) signal (Zecharia et al., 2012). HDC-Cre mice were crossed with Rosa26-loxP-Stop-loxP-YFP mice (Srinivas et al., 2001) or a floxed γ2 mouse strain (γ2I77lox), separately. The γ2I77lox line was generated with the codon of phenylalanine (F) at position 77 mutated to isoleucine (I) in exon 4 of the γ2 subunit gene, and two loxP sites inserted in intron 3 and intron 4, respectively (Wulff et al., 2007). The F77I mutation resulted in the loss of zolpidem sensitivity from all cells tested (Cope et al., 2004, 2005). Importantly, the physiological properties of the GABA_A_Rs were unchanged in the F77I strain and there was no behavioral phenotype associated with this silent mutation (Cope et al., 2004, 2005). This line has been used to delete IPSCs from a number of cell types (Wulff et al., 2007, 2009a,b; Zecharia et al., 2012; Kerti-Szigeti et al., 2014; Rovó et al., 2014). To generate HDC-Δγ2 mice and littermate controls, homozygous γ2I77lox/lox mice were crossed with heterozygous γ2I77lox/+/HDC-Cre mice. The γ2I77lox mouse line was genotyped by PCR with the following primers: forward: 5′-GTCATGCTAAATATCCTACAGTGG-3′; reverse: 5′-GGATAGTGCATCA-G¬CAGACAATAG-3′ (213 bp wild-type; 250 bp floxed allele) and the HDC-Cre mouse line was genotyped using: forward: 5′-GTGTGGCTGCCCCTTCTG-CC-3′; reverse: 5′-AGCCTCACCATGGCCCCAGT-3′ (250bp).

### Immunohistochemistry

For immunohistochemical localization, mice were deeply anesthetised with sodium pentobartital (in accordance with UK Home Office guidelines) and transcardially perfused with 4% paraformaldehyde (Thermo scientific) in phosphate buffered saline (PBS; Sigma). Coronal slices were cut at a thickness of 30 μm (Leica VT1000S vibratome) and incubated in rabbit anti-GFP (1:1000; molecular probes) and mouse anti-NeuN (1:300; Millipore) antibodies overnight. Slices were then incubated for 2 h at room temperature with Alexa Fluor 488 goat anti-rabbit (1:1000; Life technologies) and Alexa Fluor 594 goat anti-mouse IgGs (1:1000; Life technologies). Slices were then mounted in Vectashield mounting medium with DAPI (H1200, Vector labs) and the resulting red, green and blue signals were imaged on a Zeiss LSM 510 CLSM microscope (Facility for Imaging by Light Microscopy, FILM, Imperial College).

### Electrophysiology and Synaptic Recording

For electrophysiological recording, mice were routinely handled to reduce stress levels and brain slices were then prepared from adult (3–6 months postnatal) mice that were killed by cervical dislocation (in accordance with UK Home Office guidelines). The slicing solution contained (in mM) the following: NaCl 125, KCl 2.5, CaCl_2_ 2, MgCl_2_ 4, NaH_2_PO_4_ 1.25, NaHCO_3_ 26, glucose 11, 1 kynurenic acid, pH 7.4, when bubbled with 95% O_2_/5% CO_2_. Slices were cut using a vibratome tissue slicer (Campden instruments) at a thickness of 250 μm and immediately transferred to a holding chamber containing slicing solution continuously bubbled with 95% O_2_/5% CO_2_. Once slicing was complete, slices were then transferred to a 37 °C heat block for 10 min, after which the slicing solution was exchanged for recording ACSF (in mM: NaCl 125; KCl 2.5; CaCl_2_ 2; MgCl_2_ 1; NaH_2_PO_4_ 1.25; NaHCO_3_ 26; and glucose 11, pH 7.4, when bubbled with 95% O_2_/5% CO_2_). The slices were subsequently incubated in the recording ACSF at room temperature for at least another 30 min before electrophysiological recordings.

Slices were visualized using a fixed-stage upright microscope (BX51W1, Olympus) fitted with a high numerical aperture water-immersion objective and a digital camera. Patch pipettes were fabricated from thick-walled borosilicate glass capillaries (1.5 mm o.d., 0.86 mm i.d., Harvard Apparatus) using a two-step vertical puller (Narishige, PC-10). Pipette resistances were typically 3–4 MΩ when back filled with internal solution. The internal solution contained (in mM) CsCl 140, NaCl 4, CaCl_2_ 0.5, HEPES 10, EGTA 5, Mg-ATP 2; the pH was adjusted to 7.3 with CsOH. Biocytin (1.5 mg/ml) was included in the pipette solution so that cell location in the slice could be confirmed. The amplifier head stage was connected to an Axopatch 700B amplifier (Molecular Devices; Foster City, CA, USA). Fine and course movement of the pipettes were controlled by micromanipulators (PatchStar, Scientifica) mounted upon a fixed platform. The amplifier current output was filtered at 10 kHz (–3 dB, 8-pole low-pass Bessel) and digitized at 20 kHz using a National Instruments digitization board (NI-DAQmx, PCI-6052E; National Instruments, Austin, TX, USA). Data acquisition was performed using WINWCP (Version 4.1.2) and WINEDR (Version 3.0.9) kindly provided by John Dempster (John Dempster; University of Strathclyde, UK).

For reconstruction of neuronal morphology from biocytin fills, the tissue was preserved in 4% paraformaldehyde for over 48 h. Paraformaldehyde was then washed off the tissue with ice cold PBS 3×, 10 min each time. Slices were then blocked and permeabilized with 0.2% Triton-X in PBS based solution at room temperature for 1–2 h. After further washing with PBS for 10 min, slices were submerged in 2 mg/ml Streptavidin, Alexa Fluor 555 Conjugate (Life Technologies) with 0.2% Triton-X for 3–4 h at room temperature. Slices were washed again in PBS (3×, 10 min each) and mounted on slides with mounting medium (H-1000, Vectashield).

### Data Analysis

Total membrane capacitance (Cm) was calculated from Cm = Q/ΔV, where Q was the charge transfer during a hyperpolarizing 10 mV step of the command voltage (ΔV). The total membrane conductance (Gm) was calculated from Gm = Iss/ΔV where Iss was the average steady-state current during the ΔV. Cells were excluded from further analysis if Gm < 1 nS as a low resting input conductance is a defining feature of thalamic interneurons. The electrode to cell series resistance (R_S_) was calculated from the relationship R_S_= ΔV/I_P_ where I_P_ was the peak of the capacitive current transient and recordings were excluded if Rs increased by >30%. Based upon biophysical and morphological criteria, a total of 47 recordings were made from thalamic relay neurons in control mice and 66 recordings were obtained from HDC-Δγ2 mice.

Spontaneous IPSCs (sIPSCs) were detected using scaled template matching and aligned on their initial rising phases. Waveform averages were constructed from sIPSCs that exhibited monotonic rises and uninterrupted decay phase. Average baseline current levels were calculated during a 10 ms epoch immediately before each detected event and the peak amplitude was determined relative to this value. The weighted decay of individual sIPSCs was calculated as the charge transfer during the baseline corrected sIPSC divided by the sIPSC peak amplitude. The increased holding current induced by DS-2 application was calculated from all-point histograms of the holding current using the fitted peak of a single Gaussian function to calculate the average amplitude of the holding current before and after DS-2 application.

### Statistical Tests

All average values represent the mean ± the standard error of the mean (SEM). Data distributions were compared using Origin 8.5 and functions were fitted to data distributions using unconstrained least-squared fitting procedures. The type of statistical test used in each experiment is specified individually.

## Results

### The Histamine Decarboxylase Gene Drives Cre Expression in the dLGN

**Figure 1:**
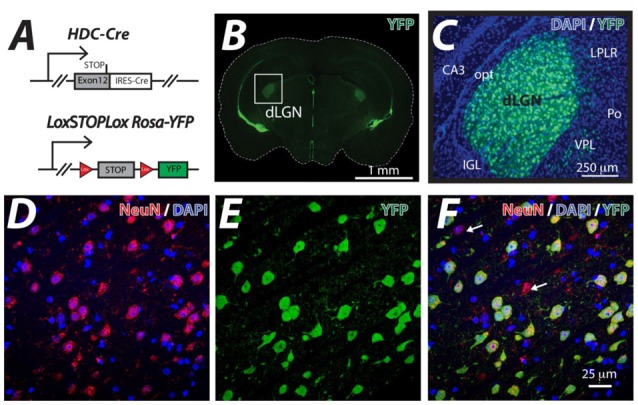
**Thalamic neuron YFP expression in the HDC-Cre mouse. (A)** The HDC-Cre line was crossed with a LoxSTOPLox Rosa-YFP mouse strain (Srinivas et al., 2001). **(B)** A brain section photographed at the level of the dorsal lateral geniculate nucleus (dLGN) showing high levels of YFP expression in the visual thalamus (boxed area) and also lining the ventricles. **(C)** A higher magnification image of YFP (green) expression observed in the visual thalamus superimposed onto the corresponding DAPI (blue) nuclear stain highlighting the CA3 region of the hippocampus. A few displaced YFP expressing cells in the adjacent Ventral PosteroLateral (VPL), posterior thalamic (Po) and the Lateral Posterior Lateral Rostral (LPLR) thalamic nuclei are shown. In contrast, no YFP expression was seen in either the optic tract (opt) or the Intergeniculate Leaflet (IGL). **(D–F)** Higher magnification confocal optical sections of the dLGN showing the results of co-fluorescent imaging of the neuron specific marker NeuN (red), DAPI (blue) and YFP (green). Note the high level of correspondence between NeuN positive neurons and YFP expression. The white arrows indicate the small number of NeuN positive neurons that do not express YFP. The larger proportion of NeuN-negative, DAPI-positive cells reflects the sizable glial cell population that is present in the dLGN.

The HDC-Cre mouse line was crossed with the LoxSTOPLox Rosa-YFP mouse line (Figure 1A) and YFP expression was examined in the resulting HDC-CRE-YFP line (Figure 1B). As expected from previous studies (Zecharia et al., 2012), the YFP signal was associated with histamine-producing neurons of the Tuberomammillary Nucleus (TMN), ependymal cells lining ventricles and putative macrophages that were sparsely distributed throughout the neocortex. However, the attention of this study was focused on the dLGN where a high proportion of cells were shown to be YFP-positive (Figure 1C). On average we found that 41% of cells were NeuN positive in the adult dLGN (DAPI+: 926 cells, NeuN+: 379 cells) counted in representative slice sections; consistent with previous estimates of neuronal density in the mammalian dLGN (Wei et al., 2011). Co-fluorescence of YFP signal with the neuronal marker NeuN indicated that YFP proteins are exclusively confined in NueN-positive neurons and ~90% of NueN-positive neurons within the dLGN had undergone recombination and expressed YFP (DAPI+: 926 cells, NeuN+: 379 cells, YFP+: 340 cells), because the hdc-cre gene is transiently expressed during postnatal development of the dLGN (Zecharia et al., 2012). In the example volume of tissue analyzed in Figure 1D, 42 out of the 47 NeuN-positive neurons (~89%) expressed YFP (Figure 1E) from a total of 102 cells that were stained with DAPI (Figures 1D–F). These results demonstrate the usefulness of the HDC-Cre mouse as a method for altering gene expression within neurons of the dLGN.

### Reduced GABAergic Drive in the γ2 Knockout

**Figure 2:**
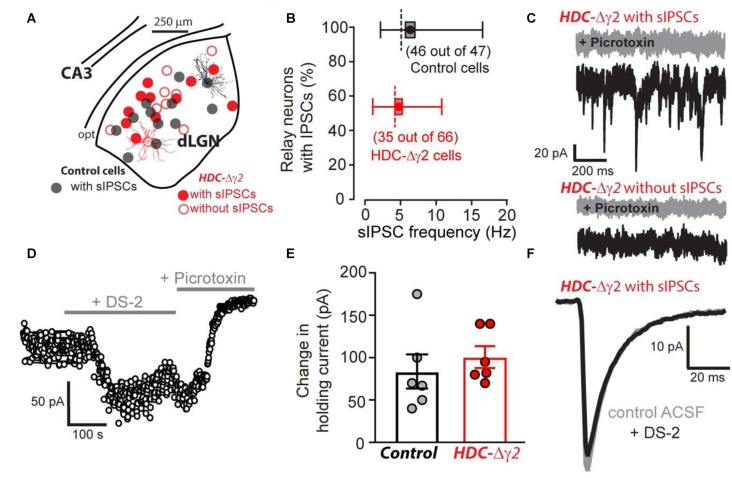
**Reduction in the occurrence of relay neurons with inhibitory postsynaptic conductance (IPSCs) within the visual thalamus of the γ2 knockout. (A)** Location of morphologically verified dLGN relay neurons that were obtained following whole-cell voltage-clamp recording in control (black filled circles) and HDC-Δγ2 mice (red open and filled circles). Note the similar locations in the dLGN of cells from both genotypes. All recordings from the control contained spontaneous IPSCs (sIPSCs). In the HDC-Δγ2, half of the cells exhibited sIPSCs (red filled circles) and half were without sIPSCs (red open circles). A reconstructed example of a full confocal projection obtained following a dLGN recording from a control cell (black trace) and an HDC-Δγ2 cell (red trace) are shown. **(B)** Box plot of sIPSC frequency distributions in 46 out of 47 dLGN control neurons (black) compared to 35 out of 66 neurons recorded from HDC-Δγ2 mice (red) that contained sIPSCs. The box shows the standard error of the mean (SEM) for the mean value, the whiskers show the 25% and 95% quartiles and the dashed line shows the mode for each distribution. **(C)** A selection of 1 s current epochs obtained during whole-cell voltage-clamp experiments at a command voltage of −60 mV. The black traces were recorded in normal solution whereas the gray sections were obtained in the presence of 30 μM picrotoxin in the extracellular solution. The top two traces were taken from an HDC-Δγ2 dLGN relay neuron that exhibited a high frequency of sIPSCs. These sIPSCs were clearly blocked by picrotoxin as was a GABA_A_ receptor (GABA_A_R)-mediated tonic conductance; as evidenced by the clear drop in steady-state holding current and the associated reduction in current noise. The lower traces were taken from an HDC-Δγ2 dLGN relay neuron that showed no evidence of sIPSCs. However, the steady-state holding current was still reduced by picrotoxin, to a similar degree as the cell above, indicating a similar level of GABA_A_R-mediated tonic conductance. **(D)** A scatter plot of the average holding current calculated during each 1 s epoch illustrating the time course of a single experiment from an HDC-Δγ2 dLGN relay neuron. Note the clear increase in the steady-state holding current observed during the application of the δ subunit-selective allosteric modulator DS-2 at a concentration of 10 μM. The subsequent application of the broad spectrum GABA_A_R antagonist picrotoxin (30 μM) into the extracellular solution reduced the steady-state holding current below control levels indicating the presence of a GABA_A_R-mediated tonic conductance. **(E)** DS-2 induced holding current change had no significant difference between control cells and HDC-Δγ2 cells (two-tailed *t* test, *P* = 0.49). **(F)** The average waveforms superimposed are taken from another HDC-Δγ2 dLGN relay neuron with remaining IPSCs, recorded in control ACSF (gray trace) and in the presence of 10 μM DS-2 (black trace). The similarity of the two superimposed average waveforms illustrates how sIPSC properties (10%–90% rise-time, peak amplitude and weighted decay) are little affected by the application of DS-2.

The HDC-Cre mouse was crossed with the γ2I77lox mouse to produce HDC-Δγ2 mice and littermate controls. Whole-cell voltage-clamp recordings were then made from identified thalamic relay neurons of the dLGN (Figure 2A). In littermate control mice, 46 out of 47 neurons exhibited sIPSCs whereas just under half of HDC-Δγ2 cells (31 out of 66) were devoid of sIPSCs (Figure 2B). Across all cells recorded, the average sIPSC frequency was 6.27 ± 0.66 Hz (*n* = 47) in control cells compared to 2.55 ± 0.4 Hz (*n* = 66) in HDC-Δγ2 cells resulting in a significant reduction (K-S test, *P* = 1 × 10^−7^) in synaptic drive following γ2 subunit removal. In those cells containing sIPSCs the average frequency was 6.41 ± 0.66 Hz (*n* = 46) in control cells compared to 4.80 ± 0.51 Hz (*n* = 31) in HDC-Δγ2 cells (K-S test, *P* = 0.21). Therefore, the main impact of γ2 removal is the loss of sIPSCs in a subset of thalamic relay neurons. As shown in Figure 2C, the remaining sIPSCs in HDC-Δγ2 cells were blocked by the GABA_A_R antagonist picrotoxin (30 μM). This blocking action was associated with a reduction in the holding current and this tonic current was observed in the control and HDC-Δγ2 neurons irrespective of the presence or absence of sIPSCs (Figure 2C). To assay any change in the contribution of δ subunit-containing GABA_A_Rs to thalamic relay neuron excitability in the HDC-Δγ2 cells, we took advantage of the allosteric modulator DS-2 (Wafford et al., 2009; Ye et al., 2013). As expected the tonic current recorded from thalamic relay neurons was enhanced by DS-2 with little action on sIPSCs (Figure 2D). The DS-2 induced change in holding current was 83.7 ± 20.1 pA (*n* = 6 cells) in control cells compared to 100.7 ± 12.9 pA (*n* = 6 cells) in HDC-Δγ2 cells with no significant difference (two-tailed *t*-test, *P* = 0.49; Figure 2E). We also did not observe any change in IPSC kinetics or amplitude during DS-2 application (Figure 2F). Consistent with no change in the tonic conductance following γ2 subunit removal, the average input conductance from the cells used in different aspects of this study was 4.03 ± 1.01 nS in the control (*n* = 47 cells from 27 mice) compared to 3.35 ± 0.89 nS (*n* = 35 cells from 16 mice) in the HDC-Δγ2 that exhibited sIPSCs and 4.14 ± 0.88 nS (*n* = 31 cells from 14 mice) in the HDC-Δγ2 that did not exhibit sIPSCs (two-tailed *t*-test, *P* > 0.44 in all cases). There was also no significant difference of membrane capacitance between these cell groups (two-tailed *t*-test, *P* > 0.12 in all cases; control cells: 99 ± 7 pF, *n* = 47; HDC-Δγ2 cells with no sIPSCs: 93 ± 5 pF, *n* = 31; HDC-Δγ2 cells with sIPSCs: 108 ± 8 pF, *n* = 35), indicating the resting membrane excitability and the cell shape had not dramatically altered. Therefore, crossing the HDC-Cre mouse line with the γ2I77lox mouse line has resulted in the complete removal of sIPSCs from only 50% of thalamic relay neurons raising the possibility that alternative synaptic GABA_A_R types contribute to phasic inhibition within the remaining cells of the dLGN.

### Fast IPSCs Are Less Prevalent in dLGN Relay Neurons of HDC-Δγ2 Mice

**Figure 3:**
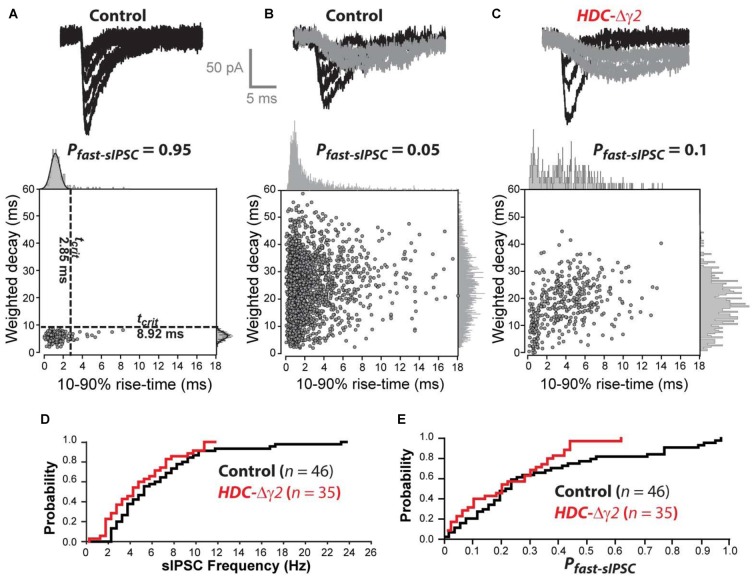
**The prevalence of fast sIPSCs is reduced in thalamic relay neurons recorded from the γ2 knockout dLGN. (A)** The top traces are superimposed individual sIPSCs recorded from a control dLGN relay neuron. The scatter plot below the traces describes the relationship between 10%–90% rise-time and weighted decay time for all sIPSCs recorded from this cell. The all-point histograms on the two axes illustrate the frequency distribution for each of these parameters. In this cell both of these distributions can be described with a single Gaussian function (solid line). The dashed lines superimposed upon the scatter plot were obtained from this Gaussian fit indicating the 95% confidence limit that defines the t_crit_ for these two parameters. These boundaries were used to define a P_fast-sIPSC_ in this cell of 0.95. **(B)** The top traces are superimposed individual sIPSCs recorded from another control dLGN relay neuron. In this example, we could identify fast rising and fast decaying sIPSCs (black traces) similar to those in **(A)** as well as slow rising and slow decaying sIPSCs (gray traces). The scatter plot below the traces in panel **(B)** describes the relationship between 10%–90% rise-time and weighted decay time for all sIPSCs recorded from this cell. The all-point histograms on the two axes illustrate the frequency distribution for each of these parameters. In this cell both of these distributions could be adequately described with a single Gaussian function. Therefore, the averaged t_crit_ values (T_crit_) obtained from the cell population illustrated in **(A)** was used to define a P_fast-sIPSC_ of 0.05 for this cell. **(C)** Similar conventions to **(B)** but the data from this cell was obtained from a HDC-Δγ2 dLGN relay neuron. **(D)** Cumulative probability distribution for all sIPSC frequency estimates for control and HDC-Δγ2 dLGN relay neurons. **(E)** Cumulative probability distribution for all P_fast-sIPSC_ estimates for control and HDC-Δγ2 dLGN relay neurons.

Kinetic analysis of sIPSCs revealed a small number of neurons in control mice (8 out of 47) that contained a single population of fast rising and fast decaying sIPSCs (Figure 3A). In two of these eight cells, we recovered fills with clear Y-like morphology similar to that reported previously for dLGN relay neurons with predominantly fast IPSCs (Bright et al., 2011). As expected from our previous studies, the majority of thalamic relay neurons (39 out of 47) exhibited a high proportion of slow rising and slow decaying sIPSCs (Figure 3B). As shown in Figure 3D, a clear reduction in sIPSC frequency was apparent across the entire population of cells with no overlap between the distributions of IPSCs in control cells and remaining IPSCs in HDC-Δγ2 cells. In order to estimate the proportion of fast sIPSCs (P_fast-sIPSC_) present in any given cell, we defined a cut-off criterion (t_crit_) for fast sIPSCs based upon data obtained from cells that exhibited a single population of fast rising and fast decaying sIPSCs. A single Gaussian fit was used to define a t_crit_ at which fast sIPSCs could be identified at a 95% confidence level. The average t_crit_ based upon Gaussian fits to the data obtained from all eight fast IPSCs-only cells (termed T_crit_, to differentiate with t_crit_ from individual cells) was 1.7 ± 0.2 ms for the rise-time and 7.9 ± 1.0 ms for the decay. Figure 3C illustrates data from an HDC-Δγ2 relay neuron that contained both fast and slow IPSCs. Using the T_crit_ values obtained from the wild-type population, the P_fast-IPSC_ was 0.1 in this cell. To determine whether γ2 deletion has reduced the prevalence of fast IPSCs across all cells, the distribution of P_fast-IPSC_ was also compared (Figure 3E). There was a reduction in the prevalence of fast IPSCs with only one recording from the HDC-Δγ2 mice giving a P_fast-IPSC_ >0.5. Indeed, γ2 subunit removal was associated with a reduction in P_fast-IPSCs_ (K-S Test, *P* < 0.001) with an average P_fast-IPSC_ of 0.27 ± 0.03 in recordings from the control mice compared to 0.11 ± 0.02 in recordings from the HDC-Δγ2 dLGN. The loss of these fast sIPSCs in the knockout may reflect the loss of γ2-containing GABA_A_Rs in a mixed GABA_A_R population in dLGN thalamic relay neurons. To test this hypothesis the pharmacological data associated with our whole-cell voltage-clamp recordings was analyzed.

### All Relay Neurons Are Affected by the γ2 Knockout

**Figure 4:**
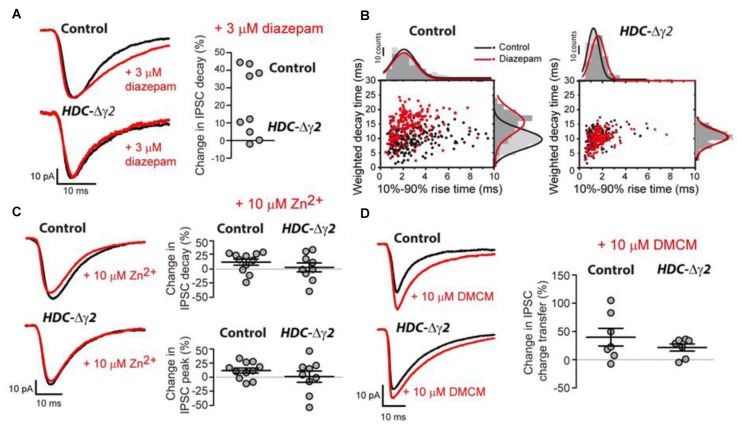
**Pharmacological evidence that the remaining sIPSCs in the γ2 knockout do not contain the γ2 subunit. (A)** Left panel: superimposed average waveforms obtained in the presence (red trace) and absence (black trace) of 3 μM diazepam in the extracellular solution. The top two waveforms were obtained from the dLGN of control mice and the bottom traces were obtained from HDC-Δγ2 mice. Right panel: scatter plot of the change in weighted decay time estimated for each cell in the control and HDC-Δγ2 dLGN. The decay of all four cells in the control dLGN was enhanced by diazepam (*n* = 4; paired *t*-test, *P* < 0.001), but there was no significant change in the decay of sIPSCs in the knockout HDC-Δγ2 dLGN (*n* = 5; paired *t*-test, *P* = 0.11). **(B)** Scatter plot of IPSC weighted decay time against 10%–90% rise-time in an example control dLGN neuron (left panel) and an HDC-Δγ2 dLGN neuron. **(C)** Similar conventions to **(A)** showing the lack of actions of 10 μM ZnCl_2_ on control and HDC-Δγ2 sIPSCs. **(D)** Similar conventions to **(A)** showing the similar actions of 10 μM methyl-6,7-dimethoxy-4-ethyl-β- carboline-3-carboxylate (DMCM) on control and HDC-Δγ2 sIPSCs. DMCM potentiated the IPSC charge transfer in both control neurons and HDC-Δγ2 dLGN neurons (Control neurons: 39.8 ± 15.59%, paired *t* test, *P* = 0.004; HDC-Δγ2 dLGN neurons: 21.57 ± 6.36%, paired *t* test, *P* = 0.03).

To distinguish between GABA_A_R heterogeneity or partial recombination in dLGN neurons, we assayed the diazepam sensitivity of the remaining sIPSCs recorded from HDC-Δγ2 neurons as the γ2F77I point mutation abolishes zolpidem sensitivity but diazepam sensitivity persists (Buhr et al., 1997; Cope et al., 2004). In the littermate control cells, 3 μM diazepam caused the average sIPSC weighed decay time to increase from 10.94 ± 0.37 ms to 15.39 ± 0.38 ms (*n* = 4; paired *t*-test, *P* < 0.001) with little change in the average 10%–90% rise-time (paired *t*-test, *P* = 0.26) or peak amplitude (paired *t*-test, *P* = 0.4) of sIPSCs. The average sIPSC waveform constructed from a control neuron before and during 3 μM illustrates this prolongation of the sIPSC decay (Figures 4A,B). 3 μM diazepam was then applied to those HDC-Δγ2 cells that contained sIPSCs. No change was observed in the average weighted decay time (*n* = 5; paired *t*-test, *P* = 0.11), 10%–90% rise-time (paired *t-test*, *P* = 0.89) or sIPSC peak amplitude (paired *t*-test, *P* = 0.27; Figures 4A,B). For example, the average weighted decay time was 10.62 ± 1.03 ms (*n* = 5) in normal ACSF vs. 11.27 ± 1.02 ms in the presence of 3 μM diazepam. The lack of diazepam sensitivity observed in the HDC-Δγ2 neurons (Figure 3B) clearly indicates that the remaining GABA_A_Rs, responsible for generating the sIPSCs, do not contain the γ2 subunit.

It is also possible that the remaining sIPSCs are meditated by αβ assemblies that lack γ subunits. However, this GABA_A_R type should be potently blocked by Zn^2+^ ions (Draguhn et al., 1990), a feature not observed in either control or HDC-Δγ2 cells (see Figure 4C). For example, in HDC-Δγ2 cells, the sIPSC peak amplitude was 93.03 ± 41.82 pA (*n* = 9) in normal ACSF compared to 90.35 ± 41.82 pA in the presence of 10 μM Zn^2+^ (paired *t*-test, *P* = 0.63) and, on average, the IPSC weighted decay time did not significantly change (+8.4 ± 6.3%, *n* = 9; paired *t*-test, *P* = 0.93). As was the case for all pharmacological manipulations described so far in this study, the sIPSC frequency remained stable at 6.2 ± 1.2 Hz in control ACSF compared to 8.1 ± 1.8 Hz in the presence of Zn^2+^ (two-tailed *t*-test, *P* = 0.2).

To test the possibility that other γ subunit-containing GABA_A_Rs contribute to the remaining IPSCs, we examined the actions of DMCM. As well as removing zolpidem sensitivity, the γ2F77I point mutation results in DMCM insensitivity (Buhr et al., 1997; Ogris et al., 2004; Leppä et al., 2005). Nevertheless, DMCM will act as an inverse agonist at γ3 subunit-containing GABA_A_Rs (Herb et al., 1992; Kerti-Szigeti et al., 2014), while DMCM will potentiate γ1-containing GABA_A_Rs (Puia et al., 1991; Khom et al., 2006; May et al., 2013). In control cells, 10 μM DMCM significantly enhanced the average sIPSC charge transfer by 39.8 ± 15.59% (*n* = 7) due to a combined action on the peak amplitude and decay of sIPSCs (paired *t*-test, *P* = 0.004; Figure 4D). A similar action of DMCM was observed in HDC-Δγ2 cells with a 21.57 ± 6.36% (*n* = 7) increase in charge transfer (paired *t*-test, *P* = 0.03; Figure 4D). These results suggest that the γ2 subunit is absent from all cells recorded from the HDC-Δγ2 dLGN, and γ1 subunit-containing GABA_A_Rs contribute to IPSCs in the dLGN.

## Discussion

We still do not fully understand the significance of GABA_A_R heterogeneity for brain function. One possibility is that the distinct kinetics conferred by different GABA_A_R subunit combinations confers flexibility to neuronal circuits that process different types of information. What is clear from this study is that the γ2 subunit is associated with fast rising and fast decaying IPSCs, whereas synaptic γ1 subunit-containing GABA_A_Rs, contribute to the slow rising and slow decaying sIPSCs within the dLGN.

### The γ1 Subunit Contributes to sIPSCs in the dLGN

The main GABA_A_R receptor genes expressed in the thalamus are α1, α4, β2 and δ (Wisden et al., 1992; Pirker et al., 2000); little γ1–3 expression is detected by either *in situ* hybridization or immunohistochemistry. Indeed, extrasynaptic GABA_A_R mediated tonic inhibition dominates in the thalamus (Jia et al., 2005; Bright et al., 2007). Nevertheless, the sensitivity of whole-cell recording is clearly able to demonstrate the presence of phasic inhibition mediated by αβγ-type synaptic receptors (Jia et al., 2005; Bright et al., 2007). We now present evidence that γ2 removal from the dLGN resulted in the complete removal of sIPSCs from half of all relay neurons and the γ2 subunit is in fact absent from the synaptic GABA_A_Rs that give rise to IPSCs in the remaining cells. This conclusion is based upon the observation that the remaining IPSCs in the γ2 knockout dLGN were diazepam insensitive (see Figures 3A,B). The diazepam induced potentiation of γ1- and γ3-containing GABA_A_Rs is much less pronounced than that known to occur at γ2-containing GABA_A_Rs (Puia et al., 1991; Herb et al., 1992; Wafford et al., 1993). The point mutation in the γ2F77Ilox line also results in DMCM insensitivity of γ2 subunit-containing GABA_A_Rs (Buhr et al., 1997). However, DMCM is an inverse agonist at γ3 subunit-containing GABA_A_Rs (Herb et al., 1992), and will potentiate currents generated by γ1 subunit-containing GABAARs (Puia et al., 1991). Therefore, the enhancement of sIPSCs we observe with DMCM (see Figure 3C) is consistent with the presence of γ1 subunit-containing GABA_A_Rs and offers a simple explanation for the sIPSCs that remain in the γ2 knockout (see Figures 4C,D). In contrast, an inhibitory action of DMCM in IPSCs of the neocortex was used to suggest that γ3 subunits are present in the synaptic GABA_A_Rs that remain following γ2 removal (Kerti-Szigeti et al., 2014). Given that only one γ subunit is present within the pentameric assembly (Olsen and Sieghart, 2009), we propose that the dLGN can express at least three distinct types of GABA_A_R. An α1, α4, β2 and δ subunit combination contributes to extrasynaptic GABA_A_Rs that mediate the tonic conductance. The α1, α4, β2 and γ2 subunit combinations will contribute to fast synaptic inhibition and we now suggest that the α1, α4, β2 and γ1 subunit combination will generate a slower form of synaptic inhibition within the dLGN.

A simple relationship between γ subunit identity and IPSC kinetics is, however, unlikely given that fast rising and fast decaying IPSCs remain in the HDC-Δγ2 dLGN neurons, possibly as a result of different GABA_A_R proximity to GABA release sites. However, our results clearly demonstrate that deletion of γ2 subunit reduced the proportion of fast rising and fast decaying IPSCs across all cells. The γ1 subunit influences GABA_A_R clustering at central synapses (Dixon et al., 2014), giving rise to slow IPSCs in neurons of the central amygdala (Esmaeili et al., 2009). Macroscopic and single channel behavior of γ1 and γ2 subunit-containing GABA_A_Rs indicates little difference in activation and deactivation, but inclusion of the γ1 subunit was reported to slow both the rise and decay of sIPSCs and this was interpreted in terms of “loose clustering” of synaptic GABA_A_Rs (Dixon et al., 2014). We have previously concluded that spillover of GABA from local dLGN interneurons did not result in the activation of high-affinity δ subunit-containing extrasynaptic GABA_A_Rs within the dLGN in spontaneous activity recordings (Bright et al., 2011; Ye et al., 2013). Consistently, we have no evidence that the sIPSCs remaining in the HDC-Δγ2 dLGN neurons involve activation of these particular receptors as the δ subunit selective drug DS-2 (Wafford et al., 2009) has no action on IPSC properties even though the tonic conductance was clearly enhanced by this allosteric modulator (see Figures 2C,D). However, spillover of GABA onto these δ subunit-containing extrasynaptic GABA_A_Rs occurs onto VB relay neurons in response to stimulated burst firing of the nRT (Herd et al., 2013). Similarly, we recently reported that DS-2 application resulted in a slowing of ChR2-evoked IPSCs that are driven by optogenetic GABA release from dLGN interneurons (Jager et al., 2016). These results are not contradictory if the magnitude of the GABA transient associated with spontaneous release were much less than the GABA transient associated with evoked release.

### Local dLGN Interneurons and the Slow sIPSC Reticular Inputs

Uniquely, GABA release within the rodent dLGN reflects afferent input from both the nRT and release from local dLGN interneurons. Other nearby first order thalamic nuclei such as the VB do not contain local interneurons, and GABA release in these nuclei is more restricted to the nRT input (Herd et al., 2013). Indeed, γ2 gene deletion from the VB nucleus resulted in a loss of IPSCs from all relay neurons examined (Rovó et al., 2014), which raises the possibility that the remaining IPSCs in the dLGN following γ2 deletion in our study are mediated by local interneurons not present in the VB. We do not exclude the possibility that the observed prevalence of the γ1 subunit can be a compensatory effect of γ2 deletion. Nonetheless, the presence of γ1-containing GABA_A_Rs following γ2 deletion in our study highlight the importance of γ subunit-containing GABA_A_Rs in the dLGN, compared to similar γ2 deletion studies mentioned above. Infrequent GABA_A_R-mediated responses did remain in some cells in Rovó et al. (2014), but the extremely slow activation/deactivation of these events was interpreted in relation to extrasynaptic δ subunit-containing GABA_A_R activation following GABA spillover. Indeed, simultaneous paired recording experiments have demonstrated that nRT burst firing can generate these slow GABA_A_R-mediated responses within VB relay neurons (Herd et al., 2013). Importantly, this particular spillover response was absent when extrasynaptic GABA_A_Rs were genetically removed. Rhythmic activity in the neocortex was little altered following γ2 deletion in VB (Rovó et al., 2014), suggesting that these spillover currents can entrain thalamocortical oscillations in the absence of fast IPSCs (Rovó et al., 2014). Previously, we have also shown that global oscillatory activity across the neocortex was not affected in the HDC-Δγ2 mice during sleep/wake cycle (Zecharia et al., 2012). The IPSCs remaining in the dLGN may well be sufficient to maintain rhythmic activity, but the presence of δ subunit-containing GABA_A_Rs may also enable spillover-mediated inhibition to occur following nRT related burst firing in a similar manner to that suggested for VB (Rovó et al., 2014).

Comparing these results with similar studies highlights the complexity of synaptic GABA_A_R targeting that is present in the mammalian brain. Purkinje cells (Wulff et al., 2009b), hippocampal parvalbumin interneurons (Wulff et al., 2009a) and VB neurons (Rovó et al., 2014) exclusively use γ2 subunit-containing GABA_A_Rs to generate fast IPSCs while some neocortical neurons make additional use of γ3 subunit-containing GABA_A_Rs to generate slow decaying IPSCs (Kerti-Szigeti et al., 2014). By combining quantitative analysis with pharmacological data in HDC-Δγ2 neurons, we have now demonstrated that deletion of γ2 subunit-containing GABA_A_Rs in the dLGN only results in complete deletion of IPSCs in half of dLGN neurons. The remaining slow rising and slow decaying IPSCs are not mediated by γ2 subunit-containing GABA_A_Rs, and they appear to involve activation of γ1 subunit-containing GABA_A_Rs. This highlights a possible requirement for distinct types of inhibitory control within the different pathways of the visual thalamus.

## Ethics Statement

This study was carried out in accordance with the recommendations of the UK Home Office and all experimental procedures have received internal approval by the Imperial College Ethical Committee and are covered by a UK Home Office License.

## Author Contributions

ZY performed electrophysiological experiments, analyzed data, prepared figures and co-wrote the manuscript. XY supervised mouse crossings and performed genotyping. CMH performed electrophysiological experiments and analyzed data. ZA performed the immunohistochemistry. NPF contributed to the writing of the manuscript. WW contributed to the writing of the manuscript. SGB analyzed data, prepared figures and wrote the manuscript.

## Funding

This work was supported by the Medical Research Council (G0901892, NPF, WW, SGB; G0800299, WW), and the Wellcome Trust (WT094211MA, SGB, WW, NPF). ZY and XY received UK/China Scholarships for Excellence PhD studentships.

## Conflict of Interest Statement

The authors declare that the research was conducted in the absence of any commercial or financial relationships that could be construed as a potential conflict of interest.
